# *In vitro* evaluation of six chemical agents on smooth *Brucella melitensis* strain

**DOI:** 10.1186/s12941-015-0077-1

**Published:** 2015-03-21

**Authors:** Zhen Wang, Peng Fei Bie, Jie Cheng, Qing Min Wu, Lin Lu

**Affiliations:** Animal Science and Technology College, Beijing University of Agriculture, Beijing, 102206 China; Key Laboratory of Animal Epidemiology and Zoonosis of the Ministry of Agriculture, College of Veterinary Medicine, China Agricultural University, Beijing, 100193 China

**Keywords:** *Brucella*, Bactericidal effect, Disinfectants, Zoonosis

## Abstract

Brucellosis is a zoonosis that disseminated by a variety of ways between animals and humans. The effective disinfection of contaminated environments, soil, feces, and animal bodies plays an irreplaceable role in the prevention and control of brucellosis. To kill *Brucella* effectively, the bactericidal effects of frequently used disinfectants (including aldehydes, halogens, quaternary ammonium compound, phenolics, and alkalines) and the potential factors that influence disinfection effects were determined in the present study. The results revealed that the minimum bactericidal concentrations (MBCs) of the six disinfectants were all significantly lower than the routinely used concentrations, and all the tested disinfectants were effective against *B. melitensis* NI. The results of quantitative determination showed that the bactericidal effects of the disinfectants were influenced by their concentration, exposure time, dirty condition and the temperature. Under dirty conditions and a low temperatures, sodium hypochlorite and sodium hydroxide showed better bactericidal effect, while benzalkonium chloride was almost without bactericidal ability. In addition, increasing the disinfectant concentration at low temperatures can improve the bactericidal effect. The present study suggested that *Brucella* is sensitive to commonly used disinfectants. However, the bactericidal effect is vulnerable to dirty conditions and low temperatures. Thus, it is necessary to test the *in vitro* sensitivity of disinfectants that are commonly used on farms or the new disinfectant formulations periodically, with the aim of improving the efficacy of animal and human brucellosis prevention programs.

## Introduction

*Brucella* spp. is a Gram-negative bacterium that is spread widely through a variety of means [1]. In domestic and wild animals, *Brucella* infection predominantly causes abortions during late gestation in females and orchitis and epididymitis in males [2]. *Brucellae* are primarily discharged into the environment via milk, vaginal discharges after abortion, and/or by urine, feces, or through the slaughtering of infected animals. *Brucella* can stay and remain active in a contaminated environment for many months, depending on conditions such as suitable temperature, pH, and humidity, [3]. Some reports have shown that *Brucella* could survive in dust, manure, water, manure slurry, aborted fetuses, soil, meat and dairy products for considerable periods of time [4]. Human and animal infections originate from direct or indirect contact with infected animals or *Brucella*-contaminated environments.

*Brucella* is known to be susceptible to heat treatment, disinfection, and direct sunlight [5]. Disinfection with chemical reagents is no doubt an important element in animal brucellosis control and eradication campaigns [6]. There are various classes of chemical disinfectants, including acids and their esters, alcohols, aldehydes, biguanides, halogens, heavy metals, oxidizing compounds, phenols and phenolic compounds, quaternary ammonium compound , quinoline and isoquinoline derivatives and dyes [7]. The selection of a disinfectant should be based on the function of disinfectant is expected to perform. All disinfectant types have advantages and disadvantages, and each has its own scope of application. For example, glutaraldehyde is a high level disinfectant for heat sensitive equipment, and which is noncorrosive to metal, and active in presence of organic material, but it is extremely irritating and toxic to skin and mucous membranes. Chlorine is a intermediate level disinfectant that used for disinfect biological material, equipment, medical supplies, and environmental surface. It is low cost, fast acting, but corrosive to metals and irritant to skin and mucous membranes [8]. The ideal disinfectant for clinical practice must fit several criteria, including water solubility, germicidal ability and cost-effectiveness according to their disinfectant targets, environments and environmental temperatures [9].

Many reports have indicated that *Brucella* species are readily killed by most of the commonly available disinfectants including hypochlorite solutions, 70% ethanol, isopropanol, iodophores, phenolic disinfectants, formaldehyde, glutaraldehyde and xylene [5,10]. However, little has been reported on the effects of the application conditions, especially with regards to low temperatures, the appropriate dosages of the disinfectants, and the organic matter that is present in dirty conditions. In the present study, *Brucella melitensis* which is the predominant strain associated with the epidemic of brucellosis in China, and six types frequently-used disinfectants in china were selected, and were evaluated in vitro for their biocidal activity on *Brucella melitensis* according different parameters: different disinfectant concentrations, contact time, temperature and in presence of different interfering conditions.

## Materials and methods

### Bacterial preparation

This study was performed with *B. melitensis* NI. This bacterium is an epidemic strain that was isolated from an aborted bovine fetus in China. This strain is referred to as smooth virulent *B. melitensis* strain biovar 3, and it induces abortion in pregnant cattle, sheep, and goats [11]. Three days prior to the study, a pure culture of *B. melitensis* NI was plated onto tryptone soya agar (TSA, BD) and incubated at 37°C with 5% (vol/vol) CO_2_ for 72 h to one week. A bacterial suspension at OD600 = 1.0 (equivalent to about 10^9^ cfu/mL) was prepared in physiological saline for the tests.

### Chemical disinfectants and their corresponding neutralizing agents

Six disinfectant types, including aldehydes, halogens, quaternary ammonium compound, phenolics, and alkalines, were selected on the basis of their active ingredients (Table 1). All disinfectants were freshly prepared according to the manufacturer's instructions prior to testing. The corresponding neutralizing agents for each disinfectant were also freshly prepared [12]. Sterile distilled water was used as a diluent and as a disinfectant control.

**Table 1:** **The disinfectants and neutralizing agents used in this study**

**Disinfectants**					**Neutralizing agents**	
**Classification**	**Active ingredients**	**Recommended concentration**	**Recommended contact time**	**Optimal application**	**Active ingredients**	**Concentration**
Aldehydes	Glutaraldehyde	4%	20 min	Equipment, goods	Glycine	2%
Halogens	Sodium hypochlorite	2 g/L	20 min	Biological material, smooth surface	Sodium thiosulfate	0.2%
	Trichloroisocyanuric acid	4 g/L	30 min	Lab environment, Medical supplies	Sodium thiosulfate	0.4%
Quaternary ammonium compound	Benzalkonium chloride	0.2 g/L	/	Skin, mucous membranes	Tween-80 + Phosphatidylcholine	0.5% + 1%
Phenolic	Lysol	10 g/L	30 min	Object surface	Tween-80	1%
Alkaline	Sodium hydroxide	10 g/L	/	Field, animal housing	Hydrochloric acid	10%

Providers for each disinfectants: Glutaraldehyde (Sinopharm Chemical Reagent Co., Ltd); Sodium hypochlorite (Beijing KeLinLong Ann medical technology co., LTD); Trichloroisocyanuric acid (Beijing ChangJiangMai Medical Technology Industry); Benzalkonium chloride (Beijing HaiDeRun Pharmaceutical CO., LTD); Lysol (Dezhou Ansett high-tech disinfection products co., LTD); Sodium hydroxide (Sinopharm Chemical Reagent Co., Ltd).

“/” indicate there is no recommended contact time.

### The MBC determination of six chemical disinfectants

Each disinfectant was diluted from the recommended concentration by a two-fold serial dilution method using sterile distilled water in test tubes. 100 μL of bacterial suspension (10^9^ cfu/mL) was added to test tubes containing the different concentrations of each disinfectant, vortexed and incubated for 20 min. The final volume of disinfectant that was added to each bacterial sample was 2 mL (100 μL of bacterial suspension with 1.9 mL of the different concentrations for each disinfectant). Sterile distilled water served as a control. After the exposure time, the test tubes were vortexed again and 100 μL of the bacterial suspension from all concentrations of each disinfectant was evenly spread over the TSA plates, each sample spread three plates. The growth was assessed after incubating for 72 h (control group) or one week (disinfection group) at 37°C with 5% CO_2_, and the minimal inhibitory concentration (MIC) value was determined. The lowest concentration at which the bacteria could not survive was recorded as MIC. Then, 0.5 mL MIC bacterial suspensions were sub-cultured in 4.5 mL liquid media without chemicals at 37°C with 5% CO_2_ to determine bacterial survival. After 72 h, 100 μL of the mixed culture were spread over a TSA plate, and the MBCs of the tested disinfectants were determined [13]. Testing was performed in triplicate. The limit of detection in this test is 7 CFU/mL.

### The bactericidal effect of chemical disinfectants under different conditions

To evaluate the influence of interfering conditions during disinfection, the bactericidal effect of each disinfectant was determined by using the MBC values in physiological saline, soil suspensions, and fecal suspensions. In brief, soil and feces were collected from a cattle shelter and made into 20% soil or 20% fecal suspensions. Samples of the soil and fecal suspensions were autoclaved at 115°C for 20 min and cooled at room temperature. The *Brucella* bacteria were then added to achieve a final bacterial concentration of 10^9^ cfu/mL. Afterward, 100 μL of bacteria suspension in physiological saline, soil and fecal suspensions were added to test tubes containing 1.9 mL of each disinfectant at the MBC concentration. Sterile distilled water served as a negative control. The exposure periods were 1 min, 5 min, and 10 min. After each exposure time, 2 mL of the corresponding neutralizing agents were added to the test tubes and neutralized for 10 min. These mixtures were 10-fold diluted and plated onto TSA to monitor the viable bacteria counts. Each sample spread three plates. The bactericidal activities of each disinfectant were expressed as logarithmic reductions in viable organisms and were calculated as follows: reduction factor (RF) = Log10 cfu (negative control)-Log10 cfu (disinfection group) [14].

### The bactericidal effect of chemical disinfectants at low temperatures

To evaluate the influence of low temperatures on the bactericidal effects, each disinfectant, bacteria suspensions in physiological saline, and soil or fecal suspensions were prepared as described above and kept on ice for 1 h. Next, 100 μL of the bacterial sample was added to test tubes containing 1.9 mL of each disinfectant at the MBC or at 2-fold the MBC. The reactions were incubated on ice for 1, 5, and 10 min [15]. After the appropriate contact time, 2 mL of the corresponding neutralizing agents were added to the test tubes and neutralized for 10 min and viable bacteria counts were monitored. The bactericidal activities of each disinfectant at different concentrations and low temperatures were calculated by employing the reduction factor [14].

### Statistical analysis

Quantitative data for the bacterial counts were expressed as the means and SD values of the Log10 reduction value (n = 3). Statistical analysis was performed by ANOVA. Significant differences were accepted at *P* < 0.05.

## Results

### The MIC and MBC for chemical disinfectants

The MIC and MBC determination results of each disinfectant are summarized in Table 2. As expected, all six disinfectants exhibited high efficacy against *B. melitensis* NI. For the aldehyde disinfectants, the MIC of glutaraldehyde was 0.125%; for halogens, 125 mg/L sodium hypochlorite and 125 mg/L trichloroisocyanuric acid were effective against *B. melitensis NI*. The MIC values for benzalkonium chloride, lysol, and sodium hydroxide were 0.002%, 0.156%, and 0.312%, respectively. In addition, the MBC determination showed that *B. melitensis* NI failed to survive after being sub-cultured in media without the tested disinfectants, a blank space suggesting that the MICs and MBCs were equivalent (Table 2).

**Table 2:** **The MBC of each disinfectant that was determined in this study**

**Disinfectant**	**Contact time**	**MIC**	**MBC**	**Recommended concentration**
Glutaraldehyde	20 min	0.125%^a^	0.125%^a^	4%
Sodium hypochlorite	20 min	125 mg/L^a^	125 mg/L^a^	2000 mg/L
Trichloroisocyanuric acid	20 min	125 mg/L^a^	125 mg/L^a^	4000 mg/L
Benzalkonium chloride	20 min	0.002%^a^	0.002%^a^	0.02%
Lysol	20 min	0.156%^a^	0.156%^a^	10%
Sodium hydroxide	20 min	0.312%^a^	0.312%^a^	10%

^a^ *P* < 0.05 (significant) in comparison with the corresponding recommended concentration.

### Bactericidal effects under different interfering conditions

**Figure 1:**
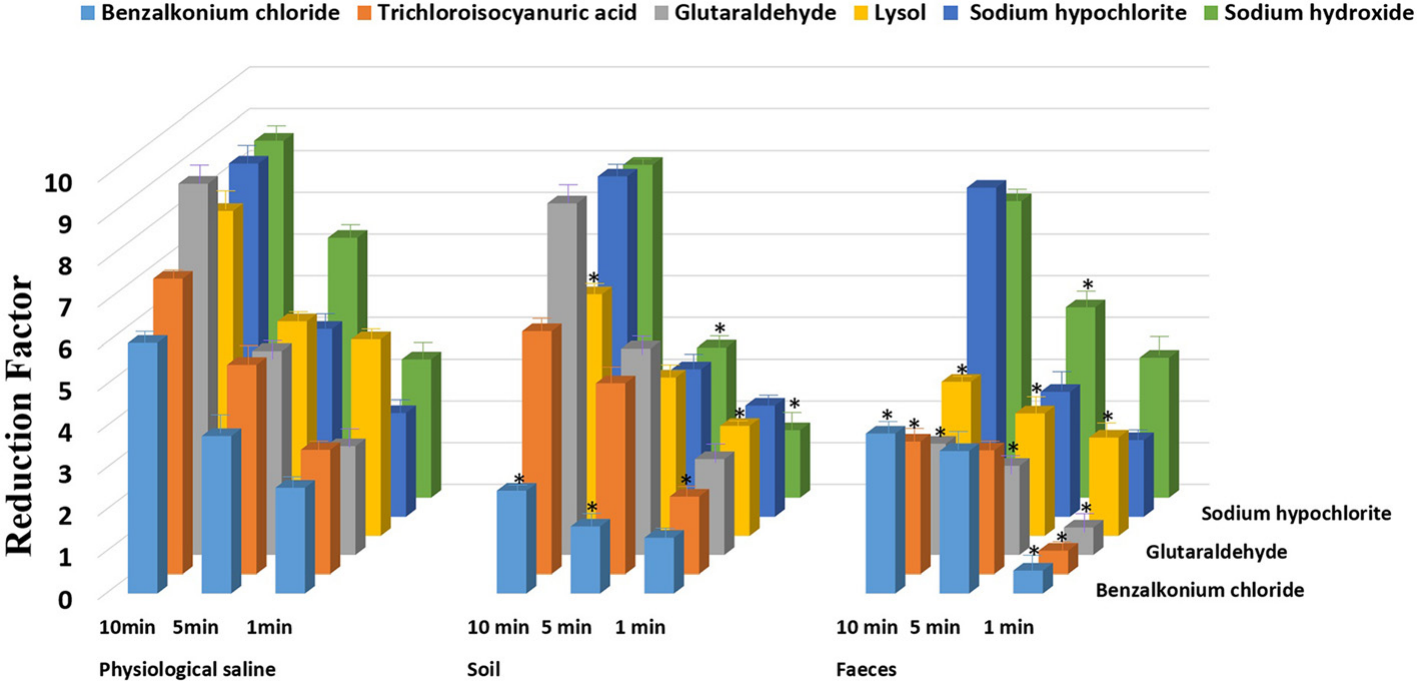
**The quantitative bactericidal effects of each disinfectant under different conditions at room temperature.** Reduction factors: The mean and SD of the Log10 cfu (negative control)-Log10 cfu (disinfection group). *P < 0.05 (significant) in comparison with the value for the physiological saline group.

The bactericidal effect of six chemical disinfectants under different conditions was determined. As shown in Figure 1, we observed that the reduction factors (RF) of glutaraldehyde, trichloroisocyanuric acid, benzalkonium chloride, and lysol in the feces suspension were significantly lower than that in physiological saline (*P* < 0.05). In the soil suspension, the RFs of these disinfectants increased with respect to that of the feces, but benzalkonium chloride still had the lowest RFs at 1.33 ± 0.23, 1.60 ± 0.08, and 2.46 ± 0.20 within 1, 5, and 10 min, respectively. Among the six disinfectants, sodium hypochlorite and sodium hydroxide were the most effective under all three conditions, with RFs of 8.47 ± 0.11 and 8.56 ± 0.21 for 10 min, respectively. In addition, the reaction time can also influence the bactericidal effects. For example, the RFs of glutaraldehyde in physiological saline were 2.61 ± 0.07, 4.89 ± 0.02, and 8.90 ± 0.14 after 1, 5, and 10 min, respectively.

### Bactericidal effects at low temperatures

**Figure 2:**
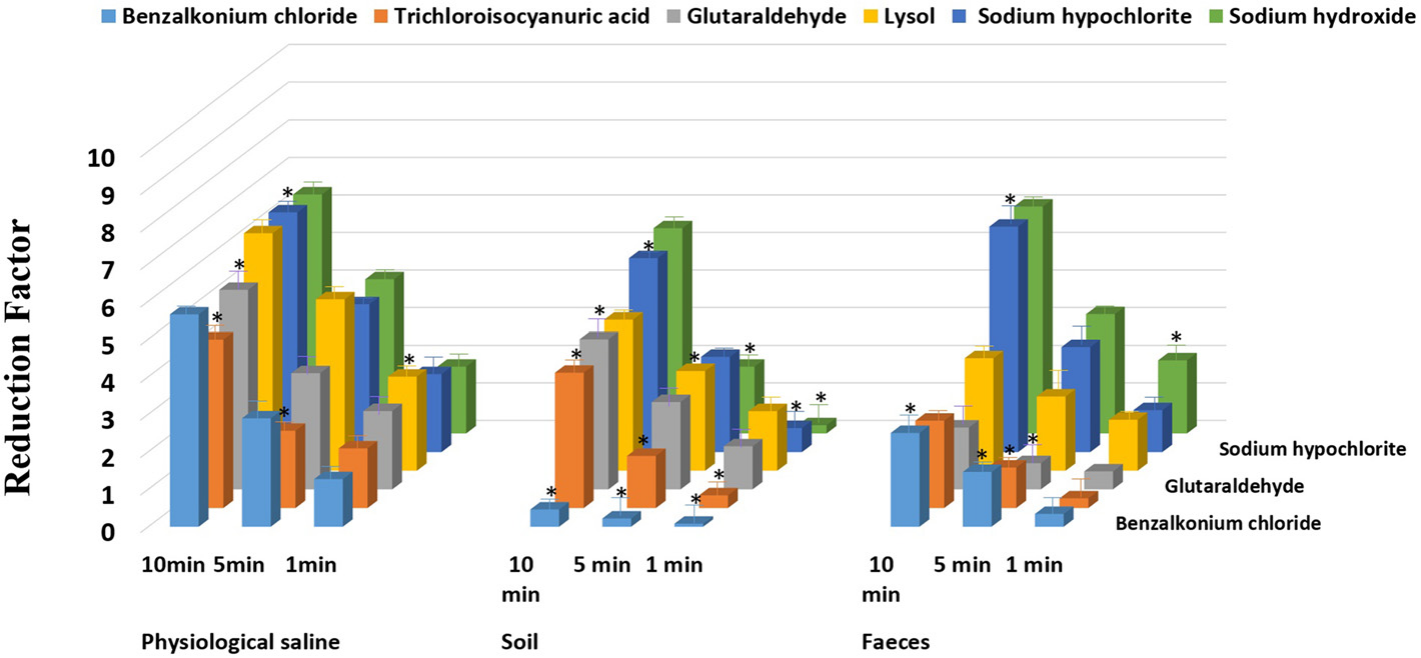
**The quantitative bactericidal effects of each disinfectant in different media at a low temperature.** Reduction factors: The mean and SD of the Log10 cfu (negative control)-Log10 cfu (disinfection group). *P < 0.05 (significant) compared with the value at room temperature.

**Figure 3:**
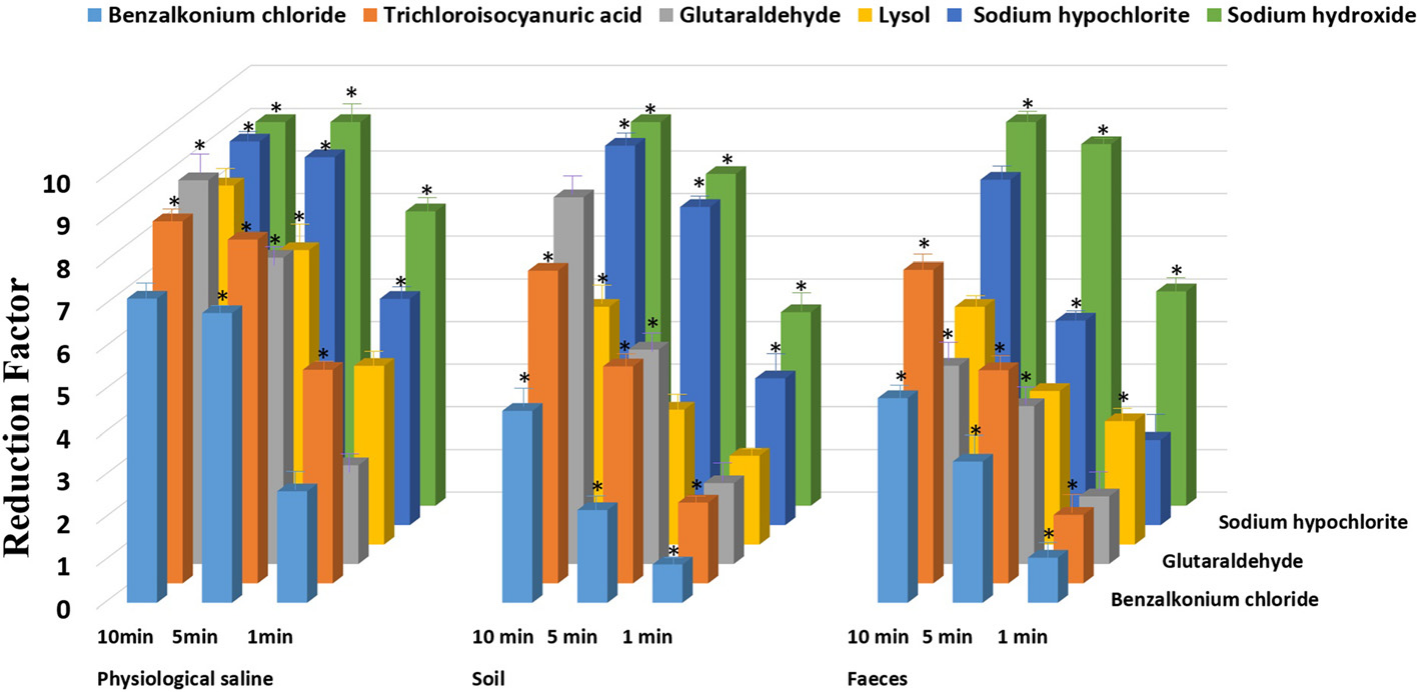
**The quantitative bactericidal effects of each disinfectant with 2-fold the MBC at a low temperature.** Reduction factors: The mean and SD of the Log10 cfu (negative control)-Log10 cfu (disinfection group). *P < 0.05 (significant) in comparison with the value at a low temperature with the MBC.

To evaluate the influence of low temperatures on the bactericidal effects of chemical disinfectants, *Brucella* bacteria samples and the tested disinfectants were cooled on ice before the test, and the reactions were also incubated on ice. As shown in Figure 2, the RFs were clearly reduced for all the disinfectants in both physiological saline and dirty conditions (*P* < 0.05). This finding was especially true for benzalkonium chloride, which almost lost its bactericidal ability with RFs of 0.08 ± 0.20, 0.22 ± 0.08, and 0.46 ± 0.18 after 1, 5, and 10 min, respectively, in the soil suspension. Moreover, the reaction time seemed to be more important under low temperatures and dirty conditions. Given a reaction time of 1 min, the RFs of sodium hypochlorite, trichloroisocyanuric acid, benzalkonium chloride, and sodium hydroxide in soil and those of glutaraldehyde, trichloroisocyanuric acid, and benzalkonium chloride in feces were all less than 1, and along with the extension in the exposure time, the RFs increased to some degree. To improve the bactericidal effect of disinfectants at low temperatures, 2-fold MBC values were used for each disinfectant. As shown in Figure 3, the RFs of the tested disinfectants at low temperatures increased and even exceeded the value found at room temperature. It is notable that the RFs of glutaraldehyde, sodium hypochlorite, and sodium hydroxide reached 9.00 for 10 min, meaning that 100% of the bacteria were killed. In addition, when maintained at a low temperature with 2-fold the MBC, sodium hypochlorite and sodium hydroxide were still the most effective.

## Discussion

Brucellosis is a highly contagious zoonosis that affects the public health and economic performance of many countries, especially in developing and underdeveloped nations, due to local husbandry methods and the lack of effective carcass and apoblema disposal, the environment was significantly contaminated with brucellosis. The pathogen, *Brucella* spp. is usually transmitted between animals through contact with contaminated environment and related products [16,17]. In addition, it was reported that *Brucellae* have been isolated from Nile catfish and rat in Egypt [18], which also indicate high threaten of brucellosis to environments. And most human cases are caused by occupational exposures to infected environments or by the ingestion of unpasteurized dairy products [5]. Disinfectants can interrupt the transmission of microorganisms and provide a public health benefit [19]. Thus, the use of effective disinfectants and appropriate disinfection measures are important process in preventing human infections and for preventing the spread of brucellosis on farms.

As previously described the disinfectant efficacy is known to depend on the concentration, time of exposure, temperature, and reaction conditions [20,21]. However, each disinfectant has its own application objectives, and disinfectant-related studies have not been adequately performed for *Brucella*. In this study, the bactericidal effects were determined for commonly used chemical disinfectants against *Brucella*. These findings indicate that the concentrations and the exposure times of these disinfectants at the determined MBC values could inhibit and kill *Brucella,* and the MBC values were much lower than the corresponding recommended concentrations (Table 2, *P* < 0.05). Although the higher concentrations may be more effective at killing pathogens, much of the compound that remains could also have side effects on the environment. Thus, it is necessary to readjust the usage concentration of disinfectants for the field disinfection process on the basis of environmental temperatures, the degree of organic matter contamination, pathogen concentration, and the disinfectants used, because the volatile and residual disinfectants may influence the environmental quality.

Although the bactericidal activity of disinfectants usually increases along with the contact time under specific conditions, liquid disinfectants can be less effective or inactivated under dirty conditions or at a low temperature [7]. Our results indicated that dirty conditions and low temperatures lowered the bactericidal effects, but higher concentrations of disinfectants and prolonged reaction times could enhance the bactericidal effects of each disinfectant on *Brucella*. Under low temperatures, the RFs of 2-fold MBCs were higher than the MBCs. These results indicated that low temperatures and concentrations influenced the bactericidal effects of the tested disinfectants to some extent. Moreover, the influence of these conditions was related to the category of disinfectants, for example, sodium hypochlorite and sodium hydroxide were barely influenced by dirty conditions and low temperatures, and the bactericidal effect of benzalkonium chloride was significantly reduced under dirty conditions and low temperatures (*P* < 0.05). Thus, it is necessary to set the guidelines for chemical disinfection use for dirty conditions especially in the fall and winter seasons.

In conclusion, given the results obtained by this study, it may be concluded that all these disinfectant types including aldehydes, halogens, quaternary ammonium compound, phenolics, and alkalines could be selected for disinfection to prevent brucellosis. Sodium hypochlorite and sodium hydroxide are preferred under dirty conditions and low temperatures. Actually, the two disinfectants are often selected on the basis of their ease of use, lower price and low toxicity [22]. In general, the present results suggested that in the process of brucellosis prevention and control, sodium hydroxide is preferred for animal housing environment and field disinfection, and sodium hypochlorite is preferred for laboratory, biological material, medical supplies, and smooth surface disinfection.
